# Statement of retraction: SLC8A1 antisense RNA 1 suppresses papillary thyroid cancer malignant progression via the FUS RNA binding protein (FUS)/NUMB like endocytic adaptor protein (Numbl) axis

**DOI:** 10.1080/21655979.2026.2730798

**Published:** 2026-10-01

We, the Editors and Publisher of the journal *Bioengineered*, have retracted the following article from publication.

Xin, Y., Shang, X., Sun, X., Xu, G., Liu, Y., & Liu, Y. (2022). SLC8A1 antisense RNA 1 suppresses papillary thyroid cancer malignant progression via the FUS RNA binding protein (FUS)/NUMB like endocytic adaptor protein (Numbl) axis. *Bioengineered*, 13(5), 12572–12582. https://doi.org/10.1080/21655979.2022.2073125

Since publication, significant concerns have been raised about the integrity of the data and reported results in the article.

When approached for an explanation, the authors provided their original data and answered our queries, but these do not sufficiently address the concerns.

As verifying the validity of published work is core to the integrity of the scholarly record, we are therefore retracting the article. The authors have been informed of this decision. The authors agree with the retraction.

We have been informed in our decision-making by our policy on publishing ethics and integrity and COPE guidelines.

The retracted article will remain online to maintain the scholarly record and will be digitally watermarked on each page as ‘Retracted’.

